# Insights on the Genetic and Phenotypic Complexities of Optic Neuropathies

**DOI:** 10.3390/genes15121559

**Published:** 2024-11-29

**Authors:** Fabiana D’Esposito, Marco Zeppieri, Maria Francesca Cordeiro, Matteo Capobianco, Alessandro Avitabile, Giuseppe Gagliano, Mutali Musa, Piero Barboni, Caterina Gagliano

**Affiliations:** 1 Imperial College Ophthalmic Research Group (ICORG) Unit, Imperial College, London NW1 5QH, UK; f.desposito@imperial.ac.uk (F.D.);; 2 Eye Clinic, Department of Neurosciences, Reproductive Sciences and Dentistry, University of Naples Federico II, 80131 Naples, Italy; 3 Department of Ophthalmology, University Hospital of Udine, Piazzale Santa Maria della Misericordia 15, 33100 Udine, Italy; 4 Western Eye Hospital, Imperial College Healthcare NHS Trust, London NW1 5QH, UK; 5 Eye Clinic, Catania University San Marco Hospital, Viale Carlo Azeglio Ciampi, 95121 Catania, Italy; 6 Department of Optometry, University of Benin, Benin City 300238, Nigeria; 7 Department of Ophthalmology, University Vita-Salute, IRCCS Ospedale San Raffaele, 20132 Milan, Italy; 8 Studio Oculistico d’Azeglio, 40123 Bologna, Italy; 9 Department of Medicine and Surgery, University of Enna “Kore”, Piazza dell’Università, 94100 Enna, Italy; 10 Mediterranean Foundation “G.B. Morgagni”, 95125 Catania, Italy

**Keywords:** optic neuropathy, genetic variants, genotype-phenotype correlations, next-generation sequencing, personalized treatment, LHON, DOA, glaucoma genetics

## Abstract

*Background*/*Objectives*: Optic neuropathies are a category of illnesses that ultimately cause damage to the optic nerve, leading to vision impairment and possible blindness. Disorders such as dominant optic atrophy (DOA), Leber hereditary optic neuropathy (LHON), and glaucoma demonstrate intricate genetic foundations and varied phenotypic manifestations. This narrative review study seeks to consolidate existing knowledge on the genetic and molecular mechanisms underlying ocular neuropathies, examine genotype-phenotype correlations, and assess novel therapeutic options to improve diagnostic and treatment methodologies. *Methods*: A systematic literature review was performed in October 2024, utilizing PubMed, Medline, the Cochrane Library, and ClinicalTrials.gov. Search terms encompassed “optic neuropathy”, “genetic variants”, “LHON”, “DOA”, “glaucoma”, and “molecular therapies”. Studies were chosen according to established inclusion criteria, concentrating on the genetic and molecular dimensions of optic neuropathies and their therapeutic ramifications. *Results*: The results indicate that DOA and LHON are mostly associated with the mitochondrial dysfunction resulting from pathogenic variants in nuclear genes, mainly *OPA1*, and mitochondrial DNA (mtDNA) genes, respectively. Glaucoma, especially its intricate variants, is linked to variants in genes like *MYOC*, *OPTN*, and *TBK1*. Molecular mechanisms, such as oxidative stress and inflammatory modulation, are pivotal in disease progression. Innovative therapeutics, including gene therapy, RNA-based treatments, and antioxidants such as idebenone, exhibit promise for alleviating optic nerve damage and safeguarding vision. *Conclusions*: Genetic and molecular investigations have markedly enhanced our comprehension of ocular neuropathies. The amalgamation of genetic and phenotypic data is essential for customized medical strategies. Additional research is required to enhance therapeutic strategies and fill the gaps in our understanding of the underlying pathophysiology. This interdisciplinary approach shows potential for enhancing patient outcomes in ocular neuropathies.

## 1. Introduction

The optic nerve is a particularly delicate structure that has the role of transmitting a sensorial visual message from the retina to the brain. Its fibers originate from the retinal ganglion cells in the retina and form the nerve itself at the level of the optic disc in the back of the eye. Optic nerves have the characteristic of a particularly high energetic demand, which is guaranteed by high mitochondrial activity [1].

Dysfunctions of the optic nerve potentially result in severe vision loss and blindness and can be related to very different causes, from developmental factors to infectious, toxic, and traumatic causes, related to myelinic degeneration, compressive, and finally genetic factors. Genetic optic nerve disorders are rare and often underdiagnosed or misdiagnosed, as the onset might be insidious and potentially confounded by different causes [2].

There are several essential diagnostic examinations that are of importance in managing and diagnosing patients. Fundus examination, a procedure using an ophthalmoscope to visualize the back of the eye, reveals bilateral and often symmetric optic disc pallor in affected patients. Gonioscopy is a diagnostic process employed to examine the anterior chamber angle of the eye, which is essential for distinguishing between open-angle and angle-closure glaucoma. Optical coherence tomography (OCT) is a non-invasive imaging modality that delivers intricate cross-sectional images of retinal layers and the optic nerve, facilitating the early identification and surveillance of optic neuropathies. Intraocular pressure (IOP), a significant risk factor for glaucoma, refers to the measurement of fluid pressure within the eye, which is crucial for disease development and treatment effectiveness. The publication offers these criteria to cater to a wider audience, encompassing academics and doctors from other disciplines [3].

The role of genetic defects in optic nerve dysfunction can be variable. Some conditions, such as Leber hereditary optic neuropathy (LHON) or dominant optic atrophy (DOA), have a definite genetic cause that is related to pathogenic variants in nuclear or mitochondrial DNA [4]. In some conditions, the genetic defect lies in genes with a pleiotropic effect, and optic neuropathy is part of a syndromic spectrum [5].

The most common optic neuropathy is glaucoma, which can be classified into different subtypes, with different levels of impact of the underlying genetic component [6]. While most forms of congenital and juvenile glaucoma are mainly Mendelian traits, the far more frequent adult glaucomas are considered genetic complex traits, wherein a particular genetic background can represent a predisposition that, when combined with other biological or epigenetic factors, can lead to the condition [7].

Comprehension of the genetic mechanisms underlying both purely genetic and genetically complex glaucomas has made enormous progress in the last decades. This is particularly related to the advances in DNA analytic techniques, with the advent of next-generation sequencing (NGS) techniques and genome-wide association studies (GWASs). NGS has allowed the analysis of large portions of DNA in a relatively short time and with gradually decreasing costs, while GWAS studies keep providing information about single-nucleotide polymorphisms (SNPs) related to an increased or decreased risk of developing glaucoma in its genetically complex forms [8].

The scope of this review is to provide an overview of current knowledge about the genetic basis of optic neuropathies, both the purely genetic ones and glaucoma, which is mainly a genetically complex trait.

## 2. Methods

A systematic literature search was performed for this narrative review in October 2024, utilizing PubMed, Medline, the Cochrane Library, and ClinicalTrials.gov. Search terms encompassed ‘optic neuropathy’, ‘genetic variations’, ‘oxidative stress’, ‘LHON’, ‘DOA’, ‘glaucoma’, and ‘molecular therapy’. Boolean operators (‘AND’, ‘OR’) were employed to enhance the specificity of the results. The preliminary search produced 372 articles, of which 39 were selected for their relevance to the issue.

Inclusion criteria were peer-reviewed publications published in English that concentrated on the genetic, molecular, or therapeutic dimensions of ocular neuropathies. Exclusion criteria encompassed those studies not pertinent to the genetic foundation, articles devoid of mechanistic specifics, or studies with insufficient data. Clinical trials were included to elucidate the translational potential of therapies aimed at genetic and molecular pathways. Updated information about genes related to optic neuropathies was obtained from both recently published papers and from Online Mendelian Inheritance in Man [9].

## 3. Genetic Foundations of Optic Neuropathies

The subsequent sections offer a comprehensive analysis of genetic optic neuropathies, emphasizing their genetic foundations, phenotypic manifestations, and contemporary treatment strategies.

### 3.1. Dominant Optic Atrophy

Dominant optic atrophy (DOA), also known as Kjer’s optic neuropathy, is the most common Mendelian inherited optic neuropathy, with an estimated prevalence of 1/30,000 in general populations and 1/10,000 in Denmark; it is primarily characterized by the bilateral degeneration of the optic nerves, leading to progressive visual loss that typically begins in the first decade of life [10]. This disease predominantly affects the retinal ganglion cells (RGCs) and their axons [11].

Usually, the disease has a slow progression and evolves into poor vision or blindness; it is associated with central or paracentral visual field deficits and a specific color vision defect called tritanopia [12]. Marked interfamilial and intrafamilial variability in the severity of symptoms is common, with visual acuity ranging from normal vision to severe visual impairment. In severe cases of DOA, visual acuity may deteriorate to the extent that patients can only detect hand movements from a short distance. The reason for this variability is not fully clear; it is probably related to a combination of environmental, genetic, and epigenetic factors [12]. Syndromic DOA (“DOA plus”) represents 20% of cases and is characterized by extra-ocular findings, such as neurosensorial hearing loss, myopathy, peripherical neuropathies, cerebellar or sensitive ataxia, and progressive ophthalmoplegia [11,13].

Despite non-syndromic DOA penetrance being around 70%, syndromic DOA presents full penetrance and usually a more severe ocular prognosis [14]. More than 70% of cases of dominant optic atrophy are due to pathogenic variants in the *OPA1* gene, which encodes a GTPase protein located in the inner mitochondrial membrane. This protein is highly expressed in the retina, as well as in the brain and muscle tissues. Mutations in *OPA1* result in haploinsufficiency, leading to mitochondrial dysfunction in the retinal ganglion cells (RGCs). RGCs are highly susceptible to ATP deficiency as they require an enormous energy demand, which is probably related to constant light exposure, leading to a particularly high level of photo-oxidative stress [15].

Most *OPA1* variants have been shown to result in dominant-negative (DN) effects, where the abnormal protein interferes with the function of the wild-type protein. Alternatively, in dominant conditions, haploinsufficiency (HI) is a mechanism wherein one normal copy of the protein is insufficient to maintain its normal cellular function [16,17].

Understanding the molecular pathogenesis of *OPA1*-linked DOA may contribute to a better understanding of other optic neuropathies. A growing body of evidence is now demonstrating the role of mitochondrial dysfunction in the neurodegenerative processes contributing to the development and progression of glaucoma [18].

Other loci and genes have been identified as responsible for optic atrophy, either as isolated forms or in conjunction with extraocular abnormalities, as summarized in Table 1 [9].

On fundus examination, the affected patients presented bilateral and often symmetric optic disc pallor, either on the temporal side or totally. OCT can quantify the fiber layer damage and the visual field examination shows central or paracentral scotoma and sparing of the peripheral visual field [11,12,19].

Currently, there is no approved treatment available for dominant optic atrophy. However, patients with significant vision loss may find low-vision aids helpful.

### 3.2. Leber Hereditary Optic Neuropathy

LHON is the most prevalent optic neuropathy; it is caused by primary mutations in mitochondrial DNA (mtDNA). It is maternally inherited and predominantly affects males, accounting for 80–90% of cases, with symptom onset usually beginning in the second or third decades of life [20]. In 95% of cases, LHON is caused by point mutations in the mtDNA genes encoding for complex I subunits, such as G3460A, G11778A, and T14484C. As in dominant optic atrophy, retinal ganglion cells seem to be selectively vulnerable to mitochondrial abnormalities [21].

LHON typically presents as painless, subacute central vision loss in one eye, with the second eye being affected weeks to months later, usually within a median time of 6–8 weeks. Fundus examination during or before the acute phase may reveal optic disc hyperemia, peripapillary telangiectasias, vascular tortuosity, and swelling of the retinal nerve fiber layer (RNFL) around the optic disc; however, some patients may have no fundus abnormalities. Over time, optic disc pallor and cupping develop, reflecting RGC death in the chronic atrophic phase [20,22].

The clinical manifestations of LHON in individuals carrying the primary mutations display a significant difference between individuals and the sexes (with around 50% of males and 90% of females being unaffected) [23]. This is probably due to environmental factors such as smoking and complex genetic mechanisms like heteroplasmy, or in combination with the inheritance of other mtDNA polymorphisms [24,25]. The male predominance of LHON has been attributed to a proposed recessive X-linked susceptibility gene, acting in conjunction with mitochondrial mutations. Hormonal factors could also explain the sex differences in LHON as estrogens can modify mitochondrial dysfunction [20]. The treatment of LHON includes supportive measures, nutritional supplements with vitamins, brimonidine for protection against oxidative stress in RGC, and ubiquinone analogs like Idebenone, a molecule that has been shown to bypass complex I and maintain ATP production, protecting the RGC from oxidative stress damage [12,26].

### 3.3. Congenital/Juvenile Glaucoma

Primary congenital glaucoma (PCG) is a non-syndromic form of glaucoma that occurs in the first three years of life and is a major cause of childhood blindness: it can have a neonatal onset (0–1 month), infantile onset (1–24 months), or late onset (>2 years); it is named juvenile glaucoma when the onset is from 3 years to puberty [27]. The prevalence of PCG estimated in Caucasian populations is about 1/18,000, but the highest rates have been observed in Saudi Arabia and consanguineous populations. Symptoms include crying, eye rubbing, or tearing, and frequently reported signs are enlargement of the eye, corneal edema, and Descemet’s fractures, known as Haab’s striae [28]. Diagnosis must be confirmed by measuring the IOP, identifying optic disc cupping, and seeing abnormalities during gonioscopy, like trabeculodysgenesis or the anterior insertion of the iris [29].

PCG is caused by the incomplete development of the trabecular meshwork and is strongly associated with genetic factors, although sporadic forms make up 60% of the total cases [30]. Pathogenic variants in four genetic loci have been associated with PCG, all following a recessive inheritance with incomplete penetrance: GLC3A, GLC3B, GLC3C, and GLC3D.

Locus GLC3A contains the cytochrome P4501B1 (*CYP1B1*) gene and is the most common gene related to PCG. In a minority of cases, it can also be linked to other phenotypes, like open-angle glaucoma, aniridia, Peters anomaly, or Axenfeld–Rieger syndrome. CYP1B1 is an enzyme that is expressed in extra-hepatic tissues and could play a crucial role in the development of the trabecular meshwork [31].

In addition, the transforming growth factor β binding protein 2 (*LTBP2*) gene is located in the GLC3D locus and its mutations are a rare cause of PCG: LTBP2 is an extracellular matrix protein involved in cell adhesion and elastin microfibril assembly [32]. Genes causing PCG have not been identified in GLC3B or GLC3C loci-related families so far [30].

In a 2016 study, Souma et al. [33] identified pathogenic variants in the *TEK* gene, encoding Tunica interna endothelial cell kinase (TEK) in some PCG patients: *TEK* mutations showed a dominant inheritance transmission pattern, with variable penetrance and expressivity. TEK is involved in vasculogenesis but is also highly expressed in the endothelium of Schlemm’s canal, although its exact role in glaucoma pathogenesis remains unclear.

PCG requires surgery in almost all cases, as medical treatments are ineffective and poorly practicable in the long term [29].

### 3.4. Glaucoma Related to Developmental Defects

Variants in a different set of genes have been detected in patients with secondary glaucomas presenting during childhood.

Specifically, *PITX2* and *FOXC1* mutations have been associated with Axenfeld–Rieger syndrome, a complex disorder with various ocular and systemic anomalies. Axenfeld–Rieger syndrome has a prevalence of 1/200,000 and a dominant inheritance, with high penetrance and variable expressivity [34].

Ocular features are usually bilateral and congenital and are caused by abnormal neural crest cell migration during the formation of ocular structures: anomalies most common reported include posterior embryotoxon, a wide range of iris and trabecular meshwork abnormalities, and ocular hypoplasia. These conditions lead to developing glaucoma in 50% of patients. Systemic features commonly reported are flat mid-facies due to maxillary hypoplasia, theets anomalies, and various heart defects like atrial septal defects [35].

*PITX2* pathogenic variants are strongly linked with Axenfeld–Rieger syndrome, as well as other developmental disorders like Peters anomaly where lens and cornea are centrally adherent: the mechanism through which defective *PITX2* leads to Axenfeld–Rieger syndrome is not fully clear, although mouse experimental studies demonstrated its importance in ocular development [36].

*FOXC1* gene pathogenetic variants are another cause of Axenfeld–Rieger syndrome: it encodes for a transcription factor crucial for cell differentiation and migration, highly expressed in eye structures like iris and trabecular meshwork. This gene has also been investigated for causing primary open-angle glaucoma in adults.

*PITX2* is a negative regulator of *FOXC1* and in this way it could in part cause Axenfeld–Rieger syndrome [30].

Another cause of developmental glaucoma is aniridia, a rare eye disease characterized by an iris development anomaly and sporadic or dominant inheritance: the disease is caused by *PAX6* mutations.

All patients have some iris tissue residues that can collapse in the iridocorneal angle, occluding aqueous humor outflow. Other vision-threatening features in aniridia are foveal hypoplasia, corneal pannus, and cataracts [37]. The *PAX6* gene encodes a transcription factor that is crucial for ocular development. Pathogenic variants of *PAX6* impair ocular development, resulting in phenotypes that are mainly characterized by aniridia, foveal hypoplasia, kerathopathy, cataracts, and glaucoma. Research utilizing animal models has demonstrated that mutations in *Pax6* disrupt downstream gene expression, which is essential for the development of the trabecular meshwork, resulting in increased intraocular pressure [38,39].

Missense variants are usually linked with the autosomal inheritance form, while sporadic cases are more often caused by deletions or rearrangements of the *PAX6* gene locus and, in some cases, may lead to WAGR syndrome (Wilms tumor, aniridia, genitourinary abnormalities, and mental retardation) or a Wilms tumor alone, due to the proximity of the *WT1* tumor suppressor gene [40].

### 3.5. Syndromic Optic Neuropathies

Optic nerve involvement is a feature of various systemic diseases and various genetic factors.

One of the best-known diseases associated with optic neuropathy is Wolfram syndrome, a rare childhood-onset disorder caused by pathogenic variants of the *WFS1* gene, characterized by optic atrophy, diabetes mellitus or insipidus, neurosensorial hearing loss, and neurological signs. The classic Wolfram syndrome has a Mendelian recessive inheritance pattern, but there is a spectrum of ‘wolframinopathies’ with different types of inheritance, like Wolfram-like syndrome (which is autosomal-dominant) [41].

The WFS1 protein, located in the endoplasmic reticulum, regulates calcium homeostasis and the unfolded protein response by binding to and degrading the ATF6 sensor, thereby preventing prolonged endoplasmic reticulum stress and cell death. Defective WFS1 proteins lead to endoplasmic reticulum dysfunction, which consequently causes optic nerve glial cell and retinal ganglion cell degeneration, in the same way as mitochondrial dysfunctions react in Leber optic neuropathy or dominant optic neuropathy [42,43].

Another genetic disorder associated with optic nerve damage is Friedreich ataxia (FRDA), a progressive neurodegenerative disorder that primarily affects the central and peripheral nervous systems, with a recessive inheritance and onset typically before the age of 25. The disease, which has an incidence of 1 in 30,000–50,000 people, is caused in 97% of cases by a homozygous GAA trinucleotide expansion in the frataxin (*FXN*) gene on chromosome 9. Pathologic *FXN* leads to frataxin deficiency, disrupting mitochondrial iron regulation and increasing susceptibility to oxidative stress: age of onset and severity of the disease depends on the length of GAA trinucleotide expansion. Clinically, FRDA presents with spinocerebellar ataxia, the loss of deep tendon reflexes, cerebellar dysarthria, and other non-neurological symptoms like cardiomyopathy, scoliosis, or diabetes. Most patients show neuro-ophthalmological signs, such as optic atrophy in 30% of cases, and eye movement abnormalities [44,45].

In 2021, Rojas P. et al. [44] described eye involvement during the progression of Friedreich ataxia: the nerve fiber layer is the first to be compromised, probably due to its richness in mitochondria. In the later stages of the disease, GCC damage, macular thinning, and optic radiation degeneration appear.

Optic nerve degeneration has also been observed also in a small percentage of patients with Charcot–Marie–Tooth disease (CMT) Type 2A, a form of heterogenous sensory motor neuropathy with genetic etiology: 11 different gene variants have been identified as pathogenetic for CMT, but the most common is the mutation of the *MFN2* gene, which encodes for a mitochondrial fusion protein [46,47,48].

## 4. Complex Traits

### 4.1. Primary Open-Angle Glaucoma

Primary open-angle glaucoma (POAG) is the most prevalent form of glaucoma, affecting 1.6% of subjects over 40 years old, and is characterized by typical glaucomatous changes in the optic nerve and other clinical features, without any identifiable secondary cause. Key risk factors for POAG include advancing age or a family history of the condition, but the most important risk factor is a high IOP, although glaucoma can also develop at normal IOP levels [6,49].

Several population studies suggest that POAG has strong genetic bases with at least 15 genes involved in pathogenesis, although DNA variants alone are not sufficient to clearly explain the development of POAG. Recent studies suggest that environmental and epigenetics factors are also involved in the pathogenesis of POAG, along with classic Mendelian inheritance mutations [50].

In total, 95% of POAG patients show a complex etiology of the disease, combining several genetic abnormalities with other risk factors. Only 5% of all POAG cases are caused by a single mutation in the myocilin (*MYOC*), optineurin (*OPTN*), or TANK binding kinase 1 (*TBK1*) gene: these three genes have a clear Mendelian dominant inheritance [6]. Pathogenic variants in genes such as *MYOC*, *OPTN*, and *TBK1* disrupt processes like protein folding, autophagy, and inflammatory regulation, which are critical for retinal ganglion cell survival. These pathways are primarily implicated in primary open-angle glaucoma (POAG) but may also overlap with the mechanisms in angle-closure glaucoma (PACG) in cases where structural anomalies exacerbate cellular dysfunction.

*MYOC* is one of the primary genes associated with primary open-angle glaucoma. Pathogenic variants in *MYOC* lead to the accumulation of abnormal proteins in the trabecular meshwork, increasing resistance to aqueous humor outflow and elevating intraocular pressure. Studies have demonstrated that pathogenetic variants of *MYOC* can influence not only the severity but also the age of onset of glaucoma, making it a significant target for genetic and therapeutic research [51].

*OPTN* (optineurin) is another gene implicated in POAG, particularly in normal-tension forms. Optineurin is a multifunctional protein that is involved in the regulation of vesicular trafficking, autophagy, and inflammatory responses. Mutations in *OPTN* can compromise these cellular functions, leading to oxidative stress and the death of RGC. Additionally, *OPTN* has been associated with various other neurodegenerative conditions like SLA, suggesting a broader role in neuronal survival and neuroinflammatory pathology.

*TBK1* (TANK-binding kinase 1) is a gene that was recently identified as a risk factor for normal-tension POAG. *TBK1* encodes a kinase that is involved in regulating the immune response and autophagy. Duplications of *TBK1* can result in the excessive activation of inflammatory pathways and altered autophagy, contributing to the degeneration of optic nerve cells [52] (Table 2).

Numerous other genes have been identified as risk factors of POAG, influencing IOP, nerve fiber cell survival, and the treatment response, and new concepts of genetic risk scores (GRS) and polygenic risk scores (PRS) are now being applied to better understand genetic data: among the most important genes, we list:

− *CAV1*/*CAV2* genes encode caveolins, the structural proteins of membrane caveolae, which regulate intraocular pressure by influencing the outflow of aqueous humor in glaucoma [53].
− *TMCO1* maintains intracellular calcium homeostasis and protects against endoplasmic reticulum stress; mutations in this gene increase intraocular pressure and the risk of optic nerve damage [54].
− *SIX1* and *SIX6* are transcription factors that are essential for eye development and the survival of retinal ganglion cells; variants in these genes may reduce the density of optic nerve fibers [54].
− *FOXC1* is associated with Axenfeld–Rieger syndrome but is also a risk factor for POAG [55].
− *CYP1B1* gene is the most common known cause of primary congenital glaucoma but, in a minority of cases, it is linked with phenotypes like open-angle glaucoma: its mutations are significant because they can disrupt the normal development of ocular tissues [55].
− *ANGPT1* encodes a protein that regulates angiogenesis and vascular stability; variants in this gene may compromise the vasculature of the optic nerve [55].
− *NTF4* produces a neurotrophic factor that supports neuronal survival, and mutations in this gene can increase the apoptosis of retinal ganglion cells in glaucoma [56,57].
− *ASB10* is involved in protein degradation in the trabecular meshwork; mutations in this gene can increase resistance to aqueous humor outflow, elevating intraocular pressure [58].
− *CDKN2A* and *CDKN2B* regulate the cell cycle and prevent apoptosis; variants in this gene can contribute to the degeneration of optic nerve cells [59].
− *WDR36* encodes a protein involved in nucleolar RNA processing and ribosome biogenesis that could potentially affect the function and survival of retinal ganglion cells [60,61].

**Table 2:** Main glaucoma-related genes.

Gene	Protein	Function	Associated Phenotype	Inheritance	Reference
*MYOC*	Myocilin	Trabecular meshwork function	Juvenile/POAG	AD	[50,51]
*TBK1*	TANK-binding kinase 1	Inflammation/autophagy	Normal-tension POAG	AD	[51,52]
*OPTN*	Optineurin	RGC survival and autophagy	Normal-tension POAG	AD	[50,51]
*CYP1B1*	Cytochrome P450 1B1	Trabecular meshwork development	Primary congenital glaucoma	AR	[31,55]

Table 2 legend. POAG: Primary open-angle glaucoma; RGC: retinal ganglion cells; AD: autosomal dominant; AR: autosomal recessive.

### 4.2. Primary Angle-Closure Glaucoma

Primary angle-closure glaucoma (PACG) is a significant cause of irreversible blindness worldwide, with a particularly high prevalence in Asian populations. Unlike POAG, which involves the gradual blockage of aqueous humor outflow, PACG is characterized by the sudden closure of the anterior chamber angle of the eye. This closure impedes the drainage of aqueous humor, leading to a rapid increase in IOP. Genetic factors are involved in the pathogenesis of primary angle-closure glaucoma by influencing ocular anatomy, extracellular matrix composition, cellular adhesion, and physiological responses [62].

Only the nanophthalmos 1 (*NNO1*) gene has been discovered to be a causative factor for a PACG phenotype, identified by the analysis of large families with nanophthalmos and hyperopia. *NNO1* pathogenic variants are associated with nanophthalmos, a condition characterized by reduced eye size and subsequent high hyperopia that predisposes individuals to PACG. The gene dysfunction itself does not directly cause PACG, but it has an effect on scleral rigidity and the ocular anatomy, increasing susceptibility to angle closure [63]. All other genes only contribute to the susceptibility of developing PACG [64].

Genetic variants in genes like *COL11A1*, which encodes a component of type XI collagen, can dictate the structural configuration of the eye, leading to a shallower anterior chamber and a thicker lens, thereby elevating the risk of angle closure [65]. Similarly, genetic variants in *PCMTD1-ST18*, *EPDR1,* and GLIS3 loci may influence eye development, affecting the positioning and size of anterior segment structures and predisposing individuals to anatomical configurations that are conducive to angle closure [66,67].

The *MMP9* gene is involved in the degradation of extracellular matrix components and some pathogenetic variants may cause scleral rigidity, increasing the risk of angle closure [68].

Another pathogenic mechanism in PACG is impaired cell adhesion between the trabecular meshwork and iris cells. Variants in genes like *PLEKHA7*, *FERMT2,* and *EPDR1*, which are essential for maintaining the junction’s adherence, can compromise the stability of the anterior chamber angle, facilitating its closure [64].

Variants in the genes regulating neurotransmitter synthesis, like *CHAT* (choline O-acetyltransferase) are identified as risk factors for PACG because they lead to abnormalities in pupil constriction and dilation that may cause the iris to bow forward (iris bombé), a well-known condition that leads to PACG [66].

Hepatocyte growth factor (*HGF*) is another gene that may play a role in regulating axial length. Variants of this gene have been associated with primary angle-closure glaucoma (PACG) and hyperopia in some populations [69].

Other potentially pathogenetic variants involve the *ABCC5* gene, but its contribution to angle-closure glaucoma is not yet fully understood. However, studies have shown that inhibiting endogenous *ABCC5* activity in zebrafish leads to a significant reduction in body length and ocular size. While some genes implicated in POAG (e.g., *CYP1B1*) also contribute to angle anomalies in PACG, the specific variants typically differ in their phenotypic expression, due to distinct anatomical and biomechanical influences.

However, without directly evidencing anatomical abnormalities, the only detection of mutations in these genes does not fully explain their involvement in the etiology of PACG [67].

### 4.3. Pseudoexfoliative Glaucoma

Pseudoexfoliative glaucoma (PEXG) is a secondary form of open-angle glaucoma that is characterized by the accumulation of fibrillar extracellular material in ocular tissues, leading to elevated IOP and optic nerve damage. The initial stage is characterized by the accumulation of extracellular fibrillar material on the ocular and surrounding tissues and is known as pseudoexfoliation syndrome (PEXS). Genetic research has significantly advanced our understanding of pseudoexfoliative glaucoma by identifying key genes involved in its pathogenesis; however, genetic factors fail to explain some aspects of the disease like its age-related onset, the asymmetric ocular manifestations, or why only some individuals with PEXS develop PEXG [70].

*LOXL1* (Lysyl oxidase-like 1) stands out as a major genetic risk factor; it encodes an enzyme involved in the formation and maintenance of elastic fibers, polymerizing tropoelastin to elastin to form elastic fibers in the extracellular matrix and cross-linking collagen and elastin fibers: two variants were found to be major risk factors across different populations, as they destabilize the extracellular matrix [71,72].

The CLU gene encodes clusterin, an extracellular chaperone protein that is involved in lipid transport, apoptosis regulation, and tissue remodeling. Zenkel et al. found the differential expression of CLU in the ocular tissues of patients with pseudoexfoliation syndrome (PEX), indicating its role in the pathological accumulation of exfoliative material [73].

Another gene that is implicated is *CACNA1A*, which encodes a subunit of the voltage-gated calcium channels involved in calcium influx and cellular functions such as neurotransmission and muscle contraction. Variants in *CACNA1A* have been associated with PEX in several populations, as they might lead to an imbalance in calcium concentrations and the formation of pseudoexfoliative aggregates [74].

Other genes recently evaluated for enhancing PEXS risk are *FBLN5* and MMPs; however, previous studies found contradictory results across different populations.

Epigenetic mechanisms are also involved in PEX pathogenesis, as they change the expression of PEXG genes. The interplay between genetic predisposition, epigenetic alterations, and environmental factors like sunlight exposure and oxidative stress contributes to the complexity of PEX pathology [74].

## 5. Molecular Mechanisms

Numerous in vitro and in vivo models have been established to investigate the effects of gene mutations on the optic nerve head and anterior chamber angle. Retinal organoids and microfluidic chip methodologies have been utilized to replicate retinal ganglion cell injury, as detailed by Su et al. (2022) [75]. Furthermore, tissue-engineered models for glaucoma research, as shown by Lu et al. (2020) [76], offer platforms to examine the implications of trabecular meshwork failure. These models facilitate the assessment of targeted therapeutics and gene-editing techniques, providing insights into disease pathogenesis and prospective treatments, integrating them into the prospective directions of future studies, which should utilize sophisticated in vitro and in vivo models to investigate the impact of genetic abnormalities across diverse cell types within the optic nerve head and anterior chamber angle. These models can connect genetic discoveries with clinical applications, facilitating innovative treatment strategies.

### 5.1. Genotype/Phenotype Correlation

Significant insights into the genotype–phenotype relationship have been made possible through the study of LHON, dominant optic atrophy (DOA), and glaucoma. In LHON, some variants are well documented, such as mitochondrial DNA mutations, in particular the m.11778G>A, m.3460G>A, and m.14484T>C variants; however, the complexity comes when we consider another factor, penetrance. Penetrance remains incomplete, and several environmental and nuclear genetic factors can modulate disease gene expression and, thus, the phenotype [77].

The genotype–phenotype correlation is similarly complex in DOA. Mutations in the *OPA1* gene correlate with a broad phenotypic spectrum, ranging from mild visual field defects to severe and irreversible optic nerve atrophy [4].

The same applies to glaucoma, where complex Mendelian and non-Mendelian genetic mechanisms coexist, with multiple loci, such as MYOC, OPTN, and TBK1, each contributing to the variability and progression of glaucomatous neurodegeneration. All these examples demonstrate how, although specific genetic mutations are often linked to characteristic phenotypes, the presence of modifying factors, such as other genes and/or environmental influences, complicates the prediction of disease severity [78,79].

This makes it clear that further research and studies are needed to understand and refine our understanding of these mechanisms and to develop appropriate diagnostic and therapeutic tools accordingly.

### 5.2. Epigenetic Mechanisms

The role of epigenetic mechanisms is one possible explanation for the variability of disease manifestations. Such mechanisms, e.g., DNA methylation, histone modification, and non-coding RNA activity, can influence the expressivity of key genes for neurodegeneration processes [80].

These are, therefore, heritable, reversible changes in gene expression that occur without altering the DNA sequence. For example, the methylation of CpG islands in the promoter regions of genes involved in mitochondrial functions can alter their expression and this, in turn, is linked to phenotypic variability in LHON and DOA. Specifically, methylation in the cytosine residues of CpG islands would appear to affect gene silencing, leading to aberrant methylation patterns that are involved in the onset and progression of neurodegeneration. In LHON, mutations in mitochondrial DNA (mtDNA) lead to defects in oxidative phosphorylation; this, as shown by recent studies, may be aggravated by nuclear DNA methylation mechanisms, which exacerbate the mitochondrial dysfunction that is already present [81,82,83].

Epigenetic mechanisms may also mediate the response to environmental stressors, such as free radical damage and reactive oxygen species, which are involved in the pathogenesis of glaucoma and LHON. Oxidative stress, in fact, can directly influence DNA methylation patterns, creating a vicious circle with the mechanisms described above. Reactive oxygen species (ROS), on the other hand, can generate dysfunctional mitochondria, induce global hypo-methylation, and promote pro-apoptotic mechanisms in retinal ganglion cells [84,85].

Other important mechanisms concern histone proteins, which are subject to a series of post-translational modifications (PTMs), such as methylation, acetylation, and phosphorylation processes. Alterations in PTMs are closely linked to optic neuropathies, altering chromatin accessibility and gene transcription, making ganglion cells more vulnerable [85,86].

For example, the reduced acetylation of histone H3 has been correlated with decreased gene expression of those genes involved in neuroprotection in glaucoma [87].

Again, in experimental models, the inhibition of histone deacetylase (HDAC) has shown protective effects on ganglion cell survival, making them potential therapeutic agents [88].

An important role is played by microRNAs (miRNAs) and long non-coding RNAs (IncRNAs), which act as critical regulators of gene expression in various neurodegenerative conditions, including optic neuropathies [89,90].

MiRNAs, small non-coding RNAs that bind to target messenger RNAs (mRNAs) to inhibit their translation, modulate retinal ganglion cell apoptosis, the oxidative stress response, and mitochondrial dynamics while lncRNAs, which can interact with chromatin editing complexes to influence gene expression, are implicated in the epigenetic regulation of neurodegenerative processes [91,92].

In glaucoma models, the dysregulation of lncRNAs has been associated with the altered expression of the genes controlling retinal ganglion cell apoptosis and stress responses [93].

Thus, epigenetic mechanisms offer a convincing explanation for the repression of phenotypic expression and the variability observed in optic neuropathies, influencing the clinical course of diseases such as LHON, DOA, and glaucoma.

### 5.3. Oxidative Stress and Mitochondria

Oxidative stress and mitochondrial dysfunction are two well-recognized elements in the pathophysiology of optic neuropathies, particularly LHON and DOA. Oxidative damage, which results from the accumulation of reactive oxygen species (ROS) as a consequence of electron transport chain (ETC) dysfunction, leads to cell damage, apoptosis, and neurodegeneration [94,95].

Recent studies show how mutations in mitochondrial DNA (mtDNA) or in the nuclear genes encoding mitochondrial proteins are decisive in the pathogenesis and progression of these diseases. For example, in LHON, mutations in ETC complex I (e.g., mutations in the MT-ND1 or MT-ND4 gene) are a paradigmatic example whereby excess ROS lead to progressive retinal ganglion cell death, resulting in optic nerve damage [96,97].

Similarly, in DOA, mutations in the OPA1 gene cause alterations in the mitochondrial fusion process, leading to mitochondrial fragmentation, increased ROS, and neuronal dysfunction. A further degree of complexity is provided by environmental factors [98].

In addition to the interaction between oxidative stress and genetically determined mitochondrial dysfunction, exposure to toxins, hypoxia, and other environmental factors can aggravate this complex mechanism. Therefore, the relationship between genotype and phenotype is again a pivotal point in the genesis and progression of neuropathies, which are complex multifactorial and multi-etiological pathologies [99].

## 6. Therapeutic Approaches

### 6.1. Molecular Therapies

The clinical implication of molecular therapies is profound, as a better understanding of these mechanisms opens the way to new therapeutic strategies based on gene modulation, the regulation of oxidative stress, and the protection of mitochondrial function [100,101,102].

In this sense, gene therapy has shown very promising results but is still in the study phase. Specifically, in the treatment of LHON, the expression of corrected copies of the ND4 gene using adeno-associated viral vectors (AAV) has led to a partial recovery of visual function in some patients carrying the m.11778G>A mutation. However, the heterogeneity in therapeutic response between patients makes it necessary to better understand the factors that modulate the efficacy of such therapy, such as the timing of the intervention and inter-individual differences in mitochondrial biology [97,103].

Other promising results come from the CRISPR/Cas9 gene editing technology, which is currently being studied to correct pathogenic DNA mutations [104].

Again, RNA-based therapies, specifically, antisense oligonucleotides, have attracted the interest of the scientific community for their ability to modulate gene expression in diseases such as DOA, where mutations in nuclear genes drive pathogenesis [10,105].

In light of the above, it is rational to think that, given the enormous genetic and molecular complexity of optic neuropathies, the most promising approach might lie in combination therapies, such as combining gene therapies with antioxidant or neuroprotective treatments. Therefore, future research should focus on the development of epigenetic therapies, such as DNA methylation inhibitors, HDAC inhibitors, and miRNA-based interventions [88,106].

### 6.2. Pharmaceutic Approaches

Understanding the key role of mitochondrial dysfunction and ROS and the use of antioxidant molecules, such as coenzyme Q10 or idebenone, is rational and has been shown to reduce oxidative stress-induced cellular damage, although clinical benefits require further confirmation through large-scale controlled studies [107,108].

Idebenone, a synthetic antioxidant with electron transport properties, was the first drug approved for the treatment of LHON in Europe. Clinical studies show how the use of this molecule with the right timing, i.e., treatment during the early stages of the disease, can slow down visual loss and, in rare cases, lead to partial visual recovery [109,110].

In the glaucoma complex, it is now well established and approved that the use of medication leads to a reduction in IOP. However, although therapies aimed at controlling IOP are effective in reducing its progression, they are not sufficient to prevent neuronal damage in all patients, which issue has pushed current research toward molecular neuroprotection strategies [111].

Again, recent studies have explored the use of kinase inhibitors, e.g., drugs that modulate the signal mediated by proteins of the kinase C group. These proteins are involved in the cellular response to mechanical and oxidative stress; therefore, inhibitors of these mechanisms have shown potential in slowing down retinal ganglion cell degeneration in animal models of glaucoma, opening up new possibilities for treatment [112,113].

Similarly, molecules that modulate apoptosis, such as BCL-2 inhibitors or other pro-apoptotic proteins, are currently being developed as potential therapies to prevent neuronal loss in degenerative optic neuropathies [114,115].

Other molecules under investigation, namely, cysteamine and nicotinamide adenine dinucleotide (NAD+), are currently being tested for their ability to support mitochondrial function and reduce the cell damage induced by metabolic and oxidative stress [116,117].

Specifically, cysteamine is used to reduce oxidative damage by increasing intracellular glutathione, a potent endogenous antioxidant; in pre-clinical studies, this molecule appears to protect retinal ganglion cells from oxidative stress. NAD+, a cofactor in redox reactions, is used to improve mitochondrial bioenergetics, a molecule of particular interest in a combination treatment approach [118,119].

## 7. Future Perspectives

The scenario of molecular defects underlying genetic optic neuropathies is particularly complex. The molecular bases of purely genetic forms, both isolated and syndromic, have been described. We have also described the genetic defects that lead to multifactorial optic neuropathies, which are known to have a strong genetic component. Novel treatments, such as gene therapy and pharmaceutical interventions, which are designed to reduce optic nerve damage and protect vision, are also described.

Our work aims at offering an overview that combines the available data, as a stimulus for additional studies on the complex genetic makeup of optic neuropathies and their phenotypes.

## Figures and Tables

**Table 1:** Genetic and phenotypic heterogeneity of optic atrophy.

Locus	MIM No.	Location	Gene	Phenotype(s) (Inheritance)
OPA1	165500	3q29	*OPA1*	Optic atrophy Optic Atrophy Plus (AD)
OPA2	311050	Xp11.4-p11.21		Optic atrophy + possible MR and ND (XL)
OPA3	165300	19q13	*OPA3*	Optic atrophy + possible cataract (AD/AR)
OPA4	605293	18q12.2-q12.3		Optic atrophy (single family linkage, AD)
OPA5	610708	12p11	*DNM1L*	Optic atrophy (AD)
OPA6	258500	8q21-q22		Optic atrophy (single family, AR)
OPA7	612988	11q14	*TMEM126A*	Optic atrophy + possible HI, CD, ND (AR)
OPA8	616648	16q21-q22		Optic atrophy, ND, HI (AD)
OPA9	616289	22q13	*ACO2*	Optic atrophy + possible ND (AD/AR)
OPA10	616732	6q21	*RTN4IP1*	Optic atrophy + possible ND (AR)
OPA11	617302	10p12	*YME1L1*	Optic atrophy (possible) + ND (AR)
OPA12	618977	18p11	*AFG3L2*	Optic atrophy + possible ND (AD)
OPA13	165510	7q34	*SSBP1*	Optic atrophy and retinal abnormalities + possible HI, PN (AD)
OPA14	620550	22q13	*MIEF1*	Optic atrophy (AD)
OPA15	620583	22q13	*MCAT*	Optic atrophy (AR)
OPA16	620629	1p35	*MECR*	Optic atrophy + HI (AR)

Table 1 Legend. MIM: Mendelian Inheritance in Man database; AD: autosomal dominant; AR: autosomal recessive; XL: X-linked; MR: mental retardation. ND: neurological disorders. HI: hearing impairment. CD: cardiac defects. PN: progressive nephropathy.

## Data Availability

Not applicable.
